# Simulation of a machine learning enabled learning health system for risk prediction using synthetic patient data

**DOI:** 10.1038/s41598-022-23011-4

**Published:** 2022-10-26

**Authors:** Anjun Chen, Drake O. Chen

**Affiliations:** LHS Technology Forum Initiative, Learning Health Community, 748 Matadero Ave, Palo Alto, CA 94306 USA

**Keywords:** Population screening, Machine learning, Translational research, Disease prevention, Health services

## Abstract

When enabled by machine learning (ML), Learning Health Systems (LHS) hold promise for improving the effectiveness of healthcare delivery to patients. One major barrier to LHS research and development is the lack of access to EHR patient data. To overcome this challenge, this study demonstrated the feasibility of developing a simulated ML-enabled LHS using synthetic patient data. The ML-enabled LHS was initialized using a dataset of 30,000 synthetic Synthea patients and a risk prediction XGBoost base model for lung cancer. 4 additional datasets of 30,000 patients were generated and added to the previous updated dataset sequentially to simulate addition of new patients, resulting in datasets of 60,000, 90,000, 120,000 and 150,000 patients. New XGBoost models were built in each instance, and performance improved with data size increase, attaining 0.936 recall and 0.962 AUC (area under curve) in the 150,000 patients dataset. The effectiveness of the new ML-enabled LHS process was verified by implementing XGBoost models for stroke risk prediction on the same Synthea patient populations. By making the ML code and synthetic patient data publicly available for testing and training, this first synthetic LHS process paves the way for more researchers to start developing LHS with real patient data.

## Introduction

As envisioned by the United States National Academy of Medicine (NAM), a learning health system (LHS) has research embedded in healthcare services, which can continuously learn and disseminate new knowledge^1–3^. Through the LHS, we aim to transform the healthcare system and engender an efficient and novel approach to innovatively solving problems that are difficult to solve with traditional approaches.

Employing machine learning (ML) of electronic health records (EHR), the concept of LHS is promising but also presents challenges^4^. Since LHS simultaneously involves system-level research and clinical practices, requirements for the initial ML models are significantly higher, and may not be met by most reported ML models using EHR. Moreover, because patient data cannot be shared openly for privacy reasons, there is a lack of openly available sets of patient data and ML code that can be used in developing and testing EHR-based ML-enabled LHS.

As a result, it is difficult for wide-spread LHS research to occur in the emerging LHS field. Since the LHS vision was first proposed in 2007, there have not yet been any reports on ML-enabled LHS implementation within a hospital clinical workflow with the key desired LHS capabilities such as automatic data collection, continuous machine learning, and rapid dissemination of new knowledge. Although several studies have reported successful applications of LHS concepts in hospital quality improvement^5,6^, these use cases did not employ continuous machine learning using EHR data.

With the advance of synthetic patient data technologies, synthetic patients have recently been accepted as alternative data for testing new processes involving EHR data^7^. It is now possible to generate synthetic patient data that can be used for developing ML-enabled LHS and be shared without restriction among research communities. Tucker et al. produced realistic synthetic data based on UK primary care patient data and tested a risk prediction algorithm for cardiovascular diseases^8^. Another study compared different approaches to generate synthetic patient data based on publicly available cancer registry data^9^.

Recently, the Synthea patient generator from mitre.org has become a common tool to generate synthetic patient data closely resembling real patient data^10^. Synthea data was validated using clinical quality measures and the synthetic patients were found to be similar to real patients with respect to these quality measures^11^. IBM researchers have encoded Synthea patient records into pathway representations for deep neural network learning, with a risk prediction accuracy of 94% in a synthetic patient population size of 500,000^12^. However, no research using Synthea data to simulate risk prediction in an ML-enabled LHS has been reported yet.

Machine learning techniques have been successfully applied on patient data, creating artificial intelligence (AI) models promising to transform health care delivery^13^. Deep patient representation using electronic health records has demonstrated the capability in predicting the future outcomes of patients^14^. Deep neural network learning of EHRs was able to unlock patient stratification at a scale suitable for personalized medicine^15^. BEHRT, a transformer for EHRs, was capable of simultaneously predicting the likelihood of 301 conditions in future visits^16,17^. Google’s EHR representation created accurate and scalable predictions for a variety of clinical scenarios from multiple health centers^18^. Benchmarks for deep learning models on large healthcare datasets were made available^19^.

Besides deep learning, there are simpler ML algorithms that are similarly effective. XGBoost is a high-performance gradient boosting algorithm^20^. XGBoost has been used to model predicted risks of many diseases, including lung cancer^21^, gastric cancer^22^, and diabetes^23^.

As ML models transition from research to clinical deployment, new challenges like reliability have emerged, triggering a paradigm shift from algorithm-centric to data-centric ML development and deployment^24,25^. The data-centric approach focuses on increasing the quality and quantity of ML-usable data by iterating through cycles of data collection, preparation, and model testing while keeping the algorithm static. In contrast, algorithm-centric ML iterates through algorithm changes while keeping data static. A successful ML program may blend both approaches in a ML pipeline to achieve the best outcomes.

We believe LHS design more closely aligns with the data-centric ML paradigm. LHS learning cycles, i.e. continuous learning from new data, improving models, and rapid dissemination to healthcare delivery, naturally provide a data-centric ML pipeline from development to deployment. Based on this new insight, we designed this LHS simulation study to demonstrate the power of data-centric LHS design using synthetic patients. The study was focused on acquiring more ML-usable data by using a basic XGBoost algorithm.

Recent reviews of cancer screening have highlighted the potential of ML-enabled LHS as the future direction of personalized cancer screening^26,27^. In collaboration with clinicians, we have been interested in applying ML-enabled LHS to increase adoption of preventive screening for disease early detection as well as reduce health care disparities in rural or otherwise underprivileged populations^28^. Because our hospital collaborators have been building LHS to personalize lung cancer and stroke screening, we chose lung cancer as an example for the development and the simulation of ML-enabled LHS and stroke for verifying the new ML-enabled LHS process. Many ML models for lung cancer^21,29,30^ and stroke^31,32^ exist and are available for comparison. A study by Kaiser Permanente used routine clinical and lab data to predict lung cancer risk^33^. Lip et al. developed gradient boosting and neural network logistic regression models that demonstrated higher performance in stroke risk prediction than current clinical rules based on decision curve analysis^34^.

Our study aimed to develop a reproducible new process using synthetic patients for building data-centric ML-enabled disease risk prediction LHS, and make the ML code with the required synthetic data for testing and training available in the LHS community. This first synthetic LHS process would have significant implications in enabling more researchers to start developing LHS with real data for solving specific problems in healthcare delivery.

## Results

This simulation study consisted of two stages: First, a new ML-enabled LHS process was developed to build a risk prediction LHS for lung cancer in synthetic patients. In the second stage, a different target disease—stroke was used to verify the effectiveness of the new LHS process for building risk prediction LHS with accurate risk prediction for any given target disease.

### Core design of ML-enabled LHS for risk prediction

**Figure 1:**
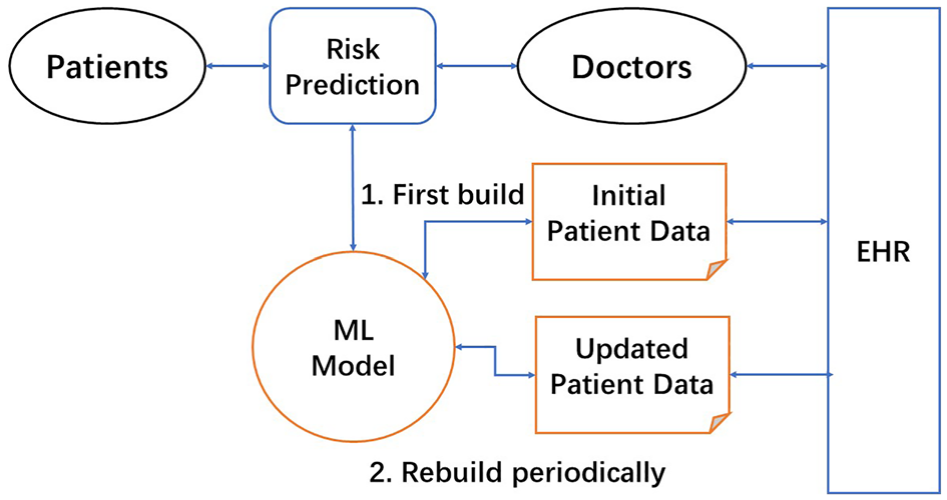
High-level diagram of data-centric and machine learning (ML) enabled learning health system (LHS) design for risk prediction. The ML model is first built with initial patient data from EHRs. LHS learning cycles continuously use updated patient data to improve the ML model, and rapidly disseminate new model for doctors to use in making risk predictions.

A simplified high-level view of the ML-enabled LHS design is shown in Fig. 1. The design is focused on the two core ML steps for simulation: (1) Build an initial ML model from existing EHR data, and (2) Continuous ML with addition of new data to improve the ML model. This LHS design essentially utilizes the data-centric ML approach for EHR patient data. Therefore, the LHS process is primarily focused on increasing the quality and quantity of ML-usable data to improve risk prediction ML models.

### Generation of Synthea synthetic patient data

Using lung cancer as the example target disease and assuming the rate of lung cancer is 0.5%, 5,000 lung cancer patients are expected in a hospital with a total of 1 million patients. In order to simulate a lung cancer risk prediction LHS at a scale of a real-world hospital EHR containing 1 million patients, the LHS should have a number of synthetic patients that corresponds to 5,000 lung cancer patients. A total of 150,000 synthetic patients were synthesized by the Synthea tool, of which ~ 5500 had lung cancer. Over 175 million points of data were available from over 13 million encounters for these Synthea patients, including 8 million diagnoses, 111 million observations, 24 million procedures and 15 million medications (see Supplementary Table S1).

### Initial ML models for lung cancer risk prediction

All records of the first dataset (pt30k) with about 30,000 Synthea patients were processed to a uniform “standard data” format. The standard data were sorted by patient and time to provide a longitudinal view of each patient’s journey. For each lung cancer target patient, data before the final diagnosis of lung cancer were collected in a patient diagnosis journey (PDJ) data profile. For each background patient (without lung cancer), a patient data profile collected data within a 40-year window starting from 30 years old. After appropriate data compression and value conversion, a single ML-ready data table was created, which contained 1,158 target patients and 29,787 background patients. The dataset was highly imbalanced, with only 3.7% positive samples. To balance the data, top background patients (with at least 100 standard data or codes) were selected to give a final ML-ready table with 1158 target patients and 4221 background patients, increasing the positive sample rate to 27.4% (Supplementary Table S2). Over 500 codes (i.e., variables) out of total ~ 750 codes were shared by the target and background patients.

**Figure 2:**
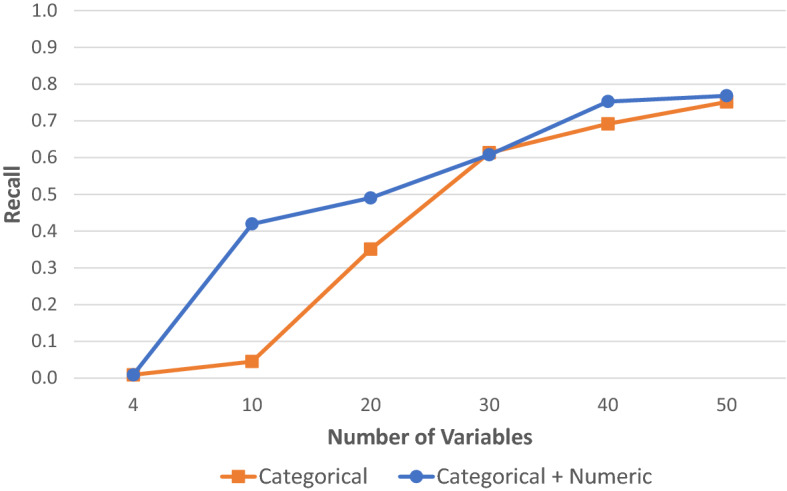
Initial tests of XGBoost model performance for lung cancer risk prediction. Prediction recall vs. number of variables used in XGBoost base models with default settings. (1) Categorical: Only categorical variables were used; (2) Categorical + Numeric: Categorical variables including continuous numeric variables that have been converted to categorical variables were used.

Initial lung cancer risk prediction (i.e., classification) models were built using the XGBoost classifier with settings specified at their defaults. The dataset was divided into 3 subsets for training, validation, and testing. The testing subset was only used for testing the model and calculating the key performance metrics: recall, precision, AUC (area under receiver operating characteristic curve) and accuracy. As shown in Fig. 2 and in Supplementary Table S3, prediction recall increased as the number of top variables increased. With 30 or more variables, precision recalls demonstrate little or no difference between categorical data alone versus a mix of categorical and continuous numeric data (after being converted to categorical data).

**Figure 3:**
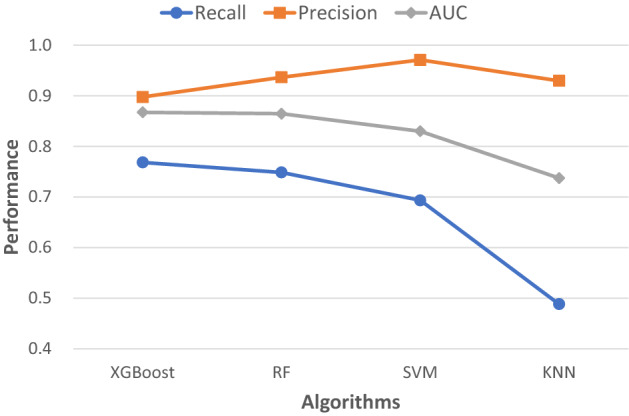
Initial performance comparison of different machine learning (ML) algorithms for lung cancer risk prediction. Base models of all algorithms used default settings of the corresponding classifiers. Model performances on a dataset of 30,000 patients and 50 variables were measured by recall, precision and AUC.

With the 30 K-patient dataset and 50 variables, the XGBoost base model was initially compared to the prediction base models of three different algorithms: Random Forest (RF), Support Vector Machines (SVM), and K-Nearest Neighbors (KNN). As shown in Fig. 3 and Supplementary Table S4, XGBoost had the best lung cancer recall and AUC measures in Synthea patients.

### Continuous improvement of lung cancer ML models in LHS

**Figure 4:**
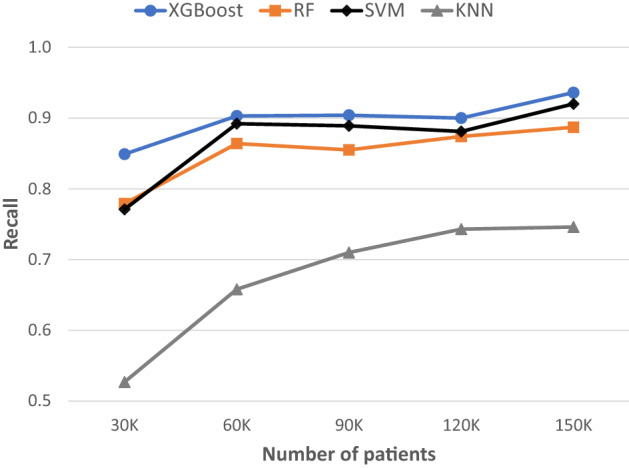
Continuous improvement of lung cancer risk prediction models with dataset size increase over time. Initial dataset: 30 K patients; 4 data updates, each with 30 K patients. Recall was used as the key performance measure for risk prediction in preventive screening. XGBoost base model was compared to the RF, SVM, and KNN base models at each instance of data update.

In the ML-enabled LHS design, new data are collected periodically and added to the last dataset to form a new updated dataset for ML. This continuous learning and improving process was simulated by adding a 30,000 Synthea patient dataset to the previous updated dataset in 4 separate instances (Supplementary Table S5). A new XGBoost model was built for each updated dataset. As shown in Table 1 and Fig. 4, as the size of dataset increased from 30,000 patients to 150,000 patients, the prediction performance of lung cancer recall increased from 0.849 to 0.936, the precision from 0.944 to 0.962, the AUC from 0.913 to 0.962, and the accuracy from 0.938 to 0.975.

**Table 1:** Continuous update of patient datasets and improvement of lung cancer risk prediction XGBoost base models in the simulated learning health system.

Dataset/metrics	Patients	Variables	XGBoost	RF	SVM	KNN
pt30k	30 K	57				
Recall			0.849	0.779	0.771	0.527
Precision			0.944	0.976	0.975	0.971
AUC			0.913	0.885	0.881	0.760
Accuracy			0.938	0.927	0.924	0.851
pt60k	60 K	83				
Recall			0.903	0.864	0.892	0.658
Precision			0.941	0.952	0.956	0.941
AUC			0.941	0.924	0.938	0.821
Accuracy			0.958	0.951	0.959	0.896
pt90k	90 K	106				
Recall			0.904	0.855	0.889	0.710
Precision			0.942	0.960	0.957	0.958
AUC			0.942	0.921	0.938	0.850
Accuracy			0.960	0.953	0.961	0.916
pt120k	120 K	119				
Recall			0.900	0.874	0.881	0.743
Precision			0.959	0.962	0.960	0.941
AUC			0.944	0.932	0.935	0.864
Accuracy			0.967	0.962	0.963	0.927
pt150k	150 K	137				
Recall			0.936	0.887	0.920	0.746
Precision			0.962	0.971	0.976	0.949
AUC			0.962	0.939	0.956	0.867
Accuracy			0.975	0.966	0.975	0.928

Initial dataset: 30 K patients; 4 data updates, each with additional 30 K patients. The XGBoost base model was also compared to RF, SVM and KNN base models at the time of each data update.

In the current literature, we could not find any study similar to our simulation study of ML-enabled synthetic data LHS. In addition, no published lung cancer risk prediction by a synthetic data ML model is available for comparison. With regard to real EMR data studies, the AUC of 0.91–0.96 for lung cancer risk prediction in this synthetic LHS study is higher than the AUC of 0.88 for predicting 1-year risk obtained in the XGBoost study by Wang et al.^21^ and AUC of 0.90 for predicting 1-year risk obtained in the deep learning study by Yeh et al.^30^.

### Comparison of different ML algorithms in risk prediction

Comparing the performance metrics of different lung cancer risk prediction models built from the 150 K-patient dataset (Table 1 and Fig. 4), XGBoost had the highest performance (recall = 0.936), followed by SVM (recall = 0.92), RF (recall = 0.887), and KNN (recall = 0.746). Since the purpose of risk prediction in the synthetic LHS was for lung cancer screening, high recall of lung cancer was considered the primary goal of the ML models. Thus, the XGBoost algorithm was selected for making lung cancer risk prediction models in the ML-enabled LHS.

### Model optimization by hyperparameter tuning

**Figure 5:**
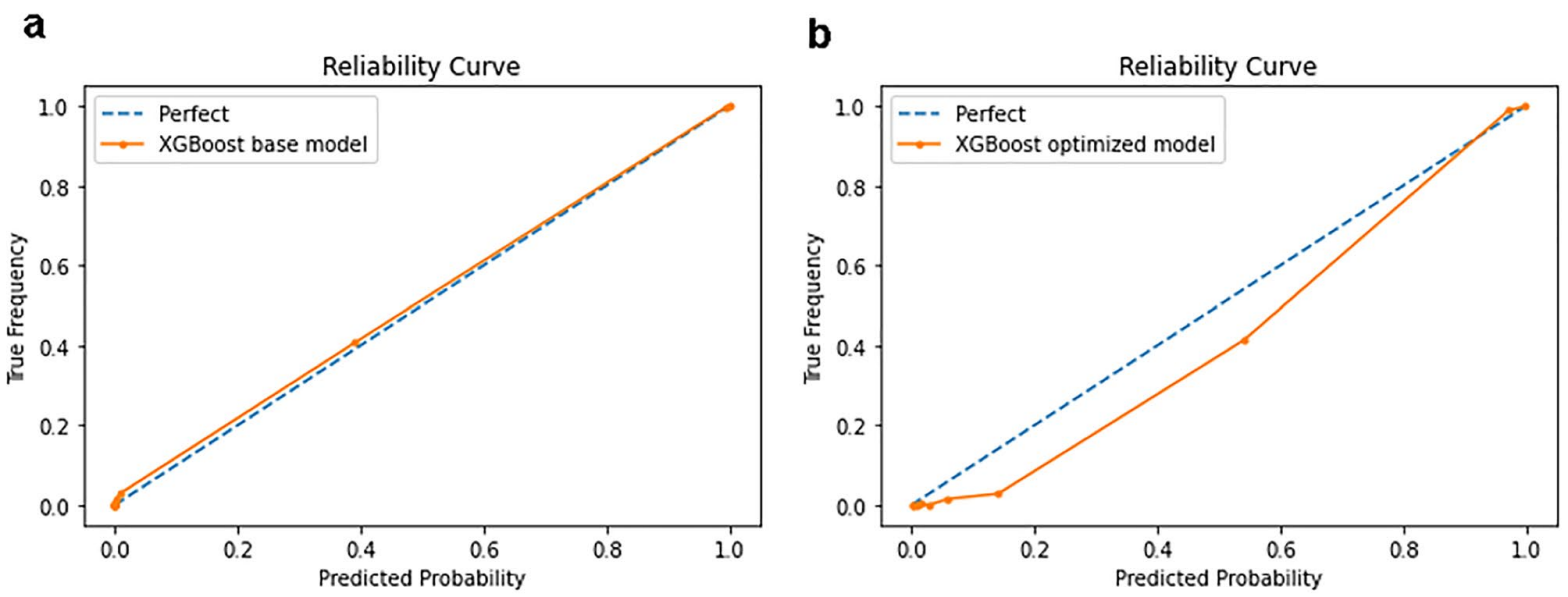
Reliability Curves of XGBoost models for lung cancer risk prediction using 150 K-patient dataset (pt150k). (**a**) Base model with default settings: recall = 0.936. (**b**) Optimized model with optimal hyperparameters: recall = 0.956.

Using a tenfold cross-validation procedure, optimal hyperparameters were found for the XGBoost optimized model. For the 30 K-patient dataset, optimization increased the prediction recall from 0.849 to 0.903. However, the optimal model’s reliability curve significantly deviated from the perfect line, indicating overfitting caused by optimization (see Supplementary Fig S2). Similarly, although the XGBoost optimized model of the pt150k dataset increased prediction recall from 0.936 to 0.956, it exhibited significant overfitting (Fig. 5b). As a result, hyperparameter tuning was not performed on the XGBoost model in the LHS learning cycles because the model’s reliability was crucial.

Instead, this LHS simulation relied on the continuous addition of data over time to iteratively improve performance of the base models. As shown in Table 1, when the dataset was updated to 150,000 patients, the prediction recall by XGBoost base model had already reached 0.936 while the model’s reliability curve was near perfect without any sign of overfitting (Fig. 5a). We expect that by increasing ML-usable data in the future rounds of data updates, the LHS would ultimately achieve the desired high performance (e.g. > 95% recall) for the XGBoost base model. Another advantage of this simpler data-centric ML process is increased ease of implementation for any hospital using this XGBoost-based risk prediction LHS with real data.

### Verification of the LHS process with stroke disease target

To verify the effectiveness of the new data-centric ML-enabled LHS established in this study, the same LHS process should be able to develop risk prediction models for any target disease such as stroke and with similar performance: high recall and precision after the same number of data update iterations.

**Figure 6:**
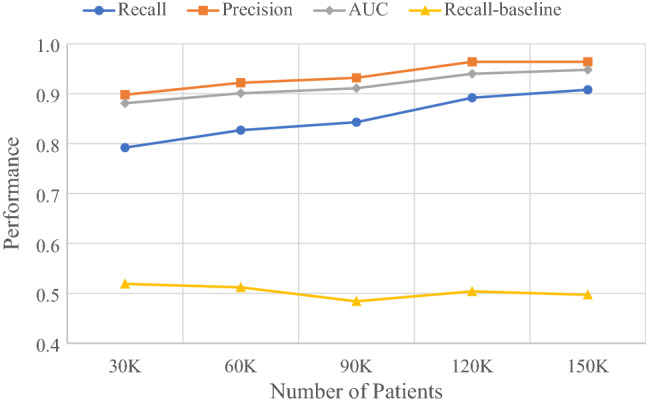
Continuous improvement of XGBoost base models for stroke risk prediction with dataset size increase over time. Model performance was measured by recall, precision and AUC. The recall of the baseline model with 10 variables is shown as reference.

Stroke occurred in Synthea patients more frequently than lung cancer. There were about 4,000 stroke patients in each of the 30 K-patient datasets. The initial stroke PDJ profile data was first prepared from the same first set of 30,000 synthetic patients (Table S7), from which the initial XGBoost base model was built for predicting stroke risk. The performance of the XGBoost base model was verified to increase as the number of its variables increased (Fig. S3). Four accumulated datasets and corresponding XGBoost models for stroke were created using the same 4 simulated datasets as in the lung cancer experiments (Table S7). As shown in Table 2 and Fig. 6, the performance metrics improved with each data update. In the fourth cycle of learning and improvement, the updated pt150k dataset had about 20,000 stroke patients and the XGBoost base model’s key metrics increased to 0.908 recall, 0.964 precision, 0.948 AUC and 0.969 accuracy.

**Table 2:** Continuous update of patient datasets and improvement of XGBoost base models for stroke risk prediction in the simulated learning health system.

Dataset/metrics	Patients	Variables	XGBoost	RF	SVM	KNN
pt30k	30 K	58				
Recall			0.792	0.708	0.711	0.626
Precision			0.898	0.920	0.938	0.906
AUC			0.881	0.844	0.848	0.802
Accuracy			0.925	0.911	0.915	0.889
pt60k	60 K	83				
Recall			0.827	0.746	0.787	0.640
Precision			0.922	0.949	0.955	0.936
AUC			0.901	0.866	0.887	0.813
Accuracy			0.938	0.925	0.936	0.898
pt90k	90 K	104				
Recall			0.843	0.760	0.807	0.646
Precision			0.932	0.958	0.961	0.944
AUC			0.911	0.875	0.898	0.817
Accuracy			0.945	0.931	0.943	0.901
pt120k	120 K	117				
Recall			0.892	0.838	0.871	0.698
Precision			0.964	0.982	0.976	0.960
AUC			0.940	0.916	0.932	0.844
Accuracy			0.964	0.955	0.962	0.916
pt150k	150 K	124				
Recall			0.908	0.855	0.888	0.720
Precision			0.964	0.987	0.977	0.957
AUC			0.948	0.925	0.940	0.855
Accuracy			0.969	0.961	0.967	0.922

Initial dataset: 30 K patients; 4 data updates, each with additional 30 K patients. The XGBoost base model was also compared to RF, SVM and KNN base models at the time of each data update.

Similar to lung cancer, there is no published result of stroke risk prediction model using Synthea data, so we compared this study’s stroke synthetic data model to the published real data models. The deep neural networks ML model in an ischemic stroke risk assessment study by Hung et al. achieved an AUC of 0.92^32^. Geisinger Health’s models using XGBoost and other common algorithms reached an AUC of 0.79 in predicting 1–5 years stroke recurrence risk^31^.

Compared to RF, SVM, and KNN algorithms in Table 2, the latest XGBoost base model (pt150k) performed the best as measured by stoke recall. The XGBoost base model’s ROC and reliability curves were in good shape (Fig. S5).

The stroke model results above confirmed that the established LHS process was similarly effective in building high-performance models for stroke risk prediction. We expect that this LHS process will be applicable to other diseases as well.

## Conclusions

In conclusion, this simulation study created the first synthetic LHS, i.e. ML-enabled learning health system of synthetic patients. It has demonstrated the effectiveness of the new LHS process, in which a risk prediction LHS can be created by building a XGBoost base model for any given target disease such as lung cancer or stroke from existing EHR data. With its intrinsically data-centric approach, the LHS can continuously learn from new patient data over time to improve the ML model performance, and ultimately achieve high recall and precision (> 95%) for disease risk prediction. The resulting synthetic data ML model cannot be used in real care delivery, so it is the LHS process that can be followed to build disease risk prediction LHS with real patient data. Finally, because real data is different from synthetic data, real data ML models can be optimized further by hyperparameter tuning.

## Discussion

Through simulating an LHS for lung cancer risk prediction and verifying its effectiveness with stroke risk prediction, the study has achieved its two goals: (1) Use synthetic patients to develop the first ML-enabled LHS that can demonstrate the effectiveness of its intrinsically data-centric approach in building high-performance risk prediction ML models for any given target disease. (2) Make the first set of synthetic ML data and code publicly available as a resource for the purpose of testing and training members of the learning health systems community.

The results of this study has two significant implications: (1) It is now feasible for hospitals to establish ML-enabled LHS for disease risk prediction in preventive screenings. (2) Because the synthetic patient data and ML code used to create the LHS in this study are publicly available, any researcher can easily obtain LHS-ready synthetic data and code to test building LHS, train LHS research teams, and ultimately guide the development of ML-enabled LHS using real EHR data.

The next step for ML-enabled LHS is validation of the new LHS process to build ML models on real patient data, which can improve many different aspects of healthcare including but not limited to risk prediction in hospitals. For example, our collaborators in hospitals are utilizing the synthetic data and ML code available from this study to develop risk prediction models for lung cancer, nasopharyngeal cancer, transient ischemic attack, and stroke using EMR-wide data of about 1 million real patients. We hope that once hospitals see the transformative benefits of the LHS approach as envisioned by the NAM^2,3^, they will implement LHS with real patient data to solve specific clinical delivery problems more effectively.

LHS have the potential to reduce health care disparities in rural and underprivileged populations. Although rural clinics and small urban community health centers (CHCs) do not have sufficient numbers of patients required for building high-accuracy ML models for various tasks by themselves, our study’s ML-enabled LHS design can be expanded to a clinical research network (CRN)^35^, called CRN-LHS. In order to enable clinics and CHCs to utilize the same ML/AI tools as big hospitals, a teaching or tertiary hospital can form a CRN with the small care providers as members and join larger LHS clinical research networks. As the center of the LHS, the primary hospital builds ML models including data from the patient populations served by the rural clinics and urban CHCs. The resulting ML models and AI tools can be rapidly disseminated among all providers within the LHS so that the small providers will not be left behind in the healthcare AI revolution. The concept of a CRN-LHS is an exciting future research direction made possible by the current LHS simulation.

This LHS simulation study focused on demonstrating the effectiveness of LHS’ intrinsically data-centric approach, but it does not mean that an algorithm-centric approach is not important. In fact, since the end-goal of LHS is successful deployment of its ML models in actual care delivery, LHS with real data may need to combine both approaches in order to reach such a difficult goal. Future studies are necessary to determine the optimal strategies for combining the data-centric and algorithm-centric approaches for developing and deploying ML models in LHS. As a general recommendation, LHS researchers using real data should consider using data-centric ML with base models to get the LHS running first, then optimize the models by hyperparameter tuning or modifying the underlying algorithms.

A myriad of efforts have aimed at sharing patient records within certain domains to enable clinical, population, and public health research after de-identification and all necessary modifications for safeguarding privacy have been performed. For example, MIMIC-III is a dataset of ~ 39,000 ICU adult patients and ~ 50,000 hospital admissions, which is freely accessible to researchers under an agreement^36^. However, many people including software developers may not meet the requirements for data access. As a result, the availability of a large ML dataset free of privacy concerns and access restrictions such as the Synthea synthetic patient data meets these requirements.

Synthea data are considered realistic but not real. They have proven useful in developing and testing ML methods or processes, but the differences between Synthea and real patient data determine where the ML models can be used. Several limitations are present in the Synthea data: the limited number of diseases, some data is biased toward certain patient populations, and some health factors such as symptoms are missing. Due to these differences, any ML model built from Synthea data cannot be used in an actual clinical setting. The risk prediction models in this simulation study are for research use only and not for real health care use.

## Methods

### Synthea patient data generation

The Synthea tool (MITRE Corp., USA) is an open-source synthetic patient generator that models the medical history of synthetic patients. Synthea was used to generate 10 populations of synthetic patients aged 30 or older, with ~ 15,000 patients per population and a total of ~ 150,000 patients. Patients were grouped into 5 datasets, of about 30,000 patients per set. Synthea patients have different domain records similar to a real EHR. including patients, encounters, diagnoses, procedures, medications, immunizations, and allergies.

A commonly available Windows laptop (Intel Core CPU 2.2 GHz, 8 GB RAM) was used for running the Synthea tool, Jupyter Notebook and machine learning Python code.

### Creation of patient standard data profiles for ML

For lung cancer as a target disease, about 5,500 patients with diagnosis of lung cancer were identified by SNOMED-CT lung cancer codes 162573006, 254637007, and 424132000 in these 150,000 synthetic patients (Supplementary Table S1). Assuming the frequency of lung cancer in the general patient population is about 0.5%, the scale of this simulation is equivalent to a hospital with 1 million patients including approximately 5,000 lung cancer patients.

Data generated by Synthea patient was coded with an international standard code. Data from all domain files in one dataset of 30,000 patients was converted to a uniformed standard data format: patient-id, time, code, name, value, unit, datatype, encounter-id, status. Standardized data was sorted by patient id and then by time in ascending order, which presented patient diagnosis journey (PDJ) over time.

In order to conduct ML on patient data for a target disease such as lung cancer risk prediction, each patient needs to have a respective data time course profile. For a target patient identified by and labeled as having lung cancer, data before the final diagnosis of lung cancer were collected and compressed to keep only the latest value for each medical code. For a background or control patient who did not have lung cancer, data within a large window between 30 and 70 years old were collected and compressed depending on the data type. For categorical data, the most common value was selected for the code of the patient, and for continuous numeric data, the average value was calculated for the patient’s code. Background patient’s age was determined by the time of last piece of data collected in the patient’s profile. Target PDJ data profiles and background standard data profiles were saved in separate files. In the 30,000 patient dataset, there were over 84,000 data in the target dataset and over 1.7 M data in the background dataset (Supplementary Table S2).

### Creation of machine learning data tables

#### ML table

Unique medical codes were collected from the above target and background data profiles separately. The total number of unique codes is over 750 while the number of overlapped codes is over 500 (Supplementary Table S2). The shared codes were used to create a single machine learning table with codes as column headers, i.e., each code representing a variable in ML. Each patient’s profile data was converted to a single row in the table, and the resulting machine learning data table has over 500 columns (variables), and a special “label” column in which the value was 1 for a target patient and 0 for a background patient.

#### Balancing data

The ML table was highly imbalanced with target patients representing about 3.6% of total patients The table was also highly sparse with most cells empty because there are over 500 variables, but most patients have only a small number of shared codes. To increase the positive sample rate, patients with at least 100 shared codes were selected to form a selected patient ML table. The resulting dataset has about 25% positive samples (see Supplementary Table S3).

#### Value conversions

Some variables and most lab tests had values in continuous numeric format and these values were converted to categorical values based on the commonly used ranges. For example, a value of < 200 mg/dL total cholesterol was converted to the category “normal” and age is converted to 2 broad categories: < 50 and ≥ 50 years old.

### Synthea patient data machine learning

#### Variable selection

Variables were sorted by the number of patients in which the variable occurred and saved in a selection file. Different numbers and types (categorical, or numeric, or both) of variables over the cutoff of 500 patients were selected for ML and comparisons.

#### Building XGBoost prediction models

XGBoost implements machine learning algorithms under the Gradient Boosting framework. XGBoost is a common ML library freely available from xgboost.readthedocs.io. XGBoost can handle missing data and thus it is optional to explicitly fill the missing data in the dataset. Python library scikit-learn from scikit-learn.org was used to perform all other ML tasks. The free Jupyter Notebook tool was used to conduct machine learning experiments. The Padas library was used to read/write csv files and manipulate data tables. Dataset was split into training, validation, and testing subsets. With default hyperparameters, XGBoost classifier was fitted with the training and validation sets and then tested by the test set independently. Model performance was measured by key metrics including recall, precision, and AUC. Because the dataset was imbalanced, the normal accuracy calculated by scikit-learn function was biased and thus not used in comparison. The ROC curve was drawn by calling scikit-learn functions.

#### Comparison of different algorithms

Scikit-learn classifiers for other common machine learning algorithms random forest, support vector machines, and k-nearest neighbors were used to build prediction models from the same dataset of 30,000 patients for comparison to the XGBoost model. Initial comparison used 50 variables and default parameters. When updated datasets became available, the same comparison was performed utilizing the updated larger datasets.

#### XGBoost model optimization

XGBoost hyperparameters are optimized by using tenfold cross validation. A range of values for key parameters are fed into the GridSearchCV() method for finding optimal values using AUC. After optimized parameters were found, optimized XGBoost models were built using these parameters. To assess the reliability of predictions after optimization, reliability curves for the base model and optimized model were plotted using scikit-learn functions and compared to one another.

### Simulation of continuous machine learning and model improvement

Four sets of about 30,000 Synthea patients were used to represent new patients collected at four different time points. At each time point, new dataset is combined with the previously updated dataset to form a new updated dataset. Thus, four updated datasets pt60k, pt90k, pt120k, and pt150k were created over time in this simulation. The resulting target and background data profile files were processed in the same way above as for the 30 K-patient dataset to ML-ready data tables.

Base models were built using the same ML process for XGBoost, RF, SVM, and KNN for comparison. As dataset size increased, using the same variable cutoff of 500 patients, more variables were selected for ML. For example, the pt150k dataset used 137 variables for ML.

### Verifying the ML-enabled LHS process with stroke

The same steps used above to create synthetic lung cancer datasets were used to create synthetic stroke datasets: create PDJ data, make an ML data table, build ML models, and run learning cycles. About 4,000 stroke patients were identified by SNOMED-CT code 230690007 in each dataset of 30,000 patients, giving a total of 20,000 stroke patients after 4 updates (Supplementary Table S6). There were 740 codes shared by stroke and non-stroke patients. Variables of all categories over the cutoff of 1000 patients were selected for ML.

## Supplementary Information

Supplementary Information.

## Data Availability

The datasets generated and analyzed during the current study are available in the Harvard Dataverse repositories. ML data and code may also be obtained from the corresponding author upon reasonable request.
