# Supplementary material for: Simulation of a machine learning enabled learning health system for risk prediction using synthetic patient data
Source: Sci Rep. 2022 Oct 26;12:17917. doi: 10.1038/s41598-022-23011-4 (PMC9606301; doi:10.1038/s41598-022-23011-4)
Supplement: Supplementary file 1 — Supplementary Information. [file 41598_2022_23011_MOESM1_ESM.pdf]

Supplementary Information

Simulation of a machine learning enabled learning health system for risk prediction using synthetic patient data

Anjun Chen, Drake Chen

Generation of Synthea patient data

The open-source Synthea tool was downloaded from https://github.com/synthetichealth/synthea. The following command was used to generate one population of synthetic adult patients of age 30 years old or older:

```
java -jar synthea-with-dependencies.jar -p 10000 -a 30-100 -s 1000 -cs 1001
```

Each population had about 15K living or deceased synthetic patients, and patient data were stored in different domain record files:

1) patients.csv
2) encounters.csv
3) conditions.csv
4) observations.csv
5) procedures.csv
6) medications.csv
7) immunizations.csv
8) allergies.csv

The total number of data records was >175M, with 111M observations, 24M procedures, 15M medications, 13M encounters, and 8M.diagnoses. The total size of files encompassing a population of 15K patients was >5GB. Ten populations were created with different seeds and grouped into 5 datasets, 30K patients per dataset. The total file size of these 150K patients was >50GB.

Preparation of Synthea patient data for lung cancer ML

Dataset	Target patients	BG patients	Encounters	Diagnoses	Observations	Procedures
pt30k	1158	29787	2733207	1738061	22276274	4880491
pt30k1	1084	29871	2704463	1731919	22101277	4828199
pt30k2	1034	29974	2720278	1748055	22315428	4898069
pt30k3	1180	30082	2716638	1748004	22362691	4872306
pt30k4	1110	29990	2721634	1749059	22388005	4894122
**Total**	**5566**	**149704**	**13596220**	**8715098**	**111443675**	**24373187**

Table S1. Five individually generated datasets of Synthea patients and record counts. Target patients: number of patients affected by the target disease (lung cancer); BG patients: number of background (unaffected) patients.

For each dataset, lung cancer PDJ data profiles and background patient data profiles were saved in separate files. Both files were joined and converted to a single ML table file after patient resampling. After value conversion, new ml table file was saved separately for comparison.

For SNOMED-CT codes, see https://browser.ihtsdotools.org/. For LOINC codes, see https://loinc.org/. For the integrated international standard codes, see UMLS Terminology Services https://uts.nlm.nih.gov/uts/.

Dataset	Target patients	BG patients	Target %	PDJ data	BG data	Selected patients	Resample Target %
pt30k	1,158	29,787	3.7	88,533	1,728,716	4,221	27.4
pt30k1	1,084	29,871	3.5	82,356	1,740,274	4,139	26.2
pt30k2	1,034	29,974	3.3	78,852	1,741,126	4,296	24.1
pt30k3	1,180	30,082	3.8	88,626	1,751,316	4,561	25.9
pt30k4	1,110	29,990	3.6	83,856	1,751,102	4,384	25.3

Table S2. Preparation of lung cancer data in five 30K-patient datasets. Target: Lung cancer: BG: background. Target %: percentage of target patients. PDJ data: number of PDJ standard data in target patients. BG data: number of standard data in background patients. Selected patients: number of patients selected after patient resampling for ML. Resample Target %: target % after resampling.

Testing variables and data types for initial lung cancer risk models

Lung cancer variables were marked for selection in the variable selection file. The open source XGBoost python library was obtained from https://xgboost.readthedocs.io/. The free Jupyter Notebook tool was obtained from https://jupyter.org/.

Variables	4	10	20	30	40	50
(1) Categorical variables only						
Recall	0.009	0.045	0.351	0.613	0.692	0.751
Precision	0.305	0.466	0.619	0.742	0.780	0.822
AUC	0.503	0.513	0.634	0.765	0.809	0.845
Accuracy	0.710	0.711	0.751	0.827	0.862	0.886
(2) Categorical + Numeric variables						
Recall	0.009	0.420	0.490	0.608	0.753	0.768
Precision	0.305	0.859	0.858	0.852	0.893	0.898
AUC	0.503	0.697	0.730	0.769	0.859	0.867
Accuracy	0.710	0.810	0.823	0.850	0.907	0.913

Table S3. Initial tests of the XGBoost base model performance for lung cancer risk prediction. Performance metrics of XGBoost base models with default settings and different numbers of variables in the first dataset of 30K patients. The 4-variable set established the baseline. (1) Only categorical variables were used. (2) Both categorical and continuous numeric variables were used after continuous numeric variables were converted to categorical.

Initial comparison of ML algorithms for lung cancer risk prediction

The Python library scikit-learn was obtained from https://scikit-learn.org/, which has implementations of the common Random Forest (RF), Support Vector Machines (SVM), and K-Nearest Neighbors (KNN) algorithms.

Metrics	XGBoost	RF	SVM	KNN
Recall	0.768	0.749	0.693	0.488
Precision	0.898	0.937	0.971	0.930
AUC	0.867	0.865	0.830	0.737
Accuracy	0.912	0.914	0.911	0.920

Table S4. Initial performance comparison of different ML algorithms for lung cancer risk prediction. Dataset: 30K patients, 50 variables. Base models for risk prediction were generated using the default settings of the corresponding classifiers.

Continuous update of datasets and lung cancer risk models

Patients and their corresponding data were added to the initial population at 4 separate time points.

Update Dataset	Target patients	BG patients	Target %	Target data	BG data	Selected patients	Resample Target %
pt30k	1158	29787	3.7	88533	1728716	4221	27.4
pt60k	2242	59658	3.6	170889	3526857	8484	26.4
pt90k	3276	89632	3.5	249741	5296989	12927	25.3
pt120k	4456	119714	3.6	338367	7077441	18209	24.5
pt150k	5566	149704	3.6	422223	8857618	22811	24.4

Table S5. Updated datasets for lung cancer risk models in the LHS. Target patient percentage increases after resampling the imbalanced datasets. Target: lung cancer. BG: background. Selected patients: patients with or without lung cancer.

Code	Characteristics	Patient Count	Patient Percentage
	All	22811	100.0%
C-424144002	Age (years)	22811	100.0%
	<50	5606	24.6%
	>=50	17205	75.4%
C-263495000	Gender	22811	100.0%
	Male	12846	56.3%
	Female	9965	43.7%
C-125680007	Marital Status	22811	100.0%
	Married	18282	80.1%
	Single	4529	19.9%
C-103579009	Race	22811	100.0%
	White	18677	81.9%
	Black	1908	8.4%
	Asian	1644	7.2%
	Hawaiian	250	1.1%
	Native	108	0.5%
	Other	224	1.0%
C-186034007	Ethnicity	22811	100.0%
	Hispanic	2512	11.0%
	Nonhispanic	20299	89.0%
C-39156-5	Body Mass Index	22798	99.9%
	Abnormal	22340	97.9%
	Normal	458	2.0%
C-72166-2	Tobacco smoking status NHIS	22798	99.9%
	Former	9716	42.6%
	Never	13082	57.3%
C-449868002	Smokes tobacco daily	882	3.9%
C-10509002	Acute bronchitis (disorder)	8693	38.1%
C-26929004	Alzheimer's disease (disorder)	245	1.1%
C-271737000	Anemia (disorder)	7509	32.9%
C-49436004	Atrial Fibrillation	1097	4.8%
C-431855005	Chronic kidney disease stage 1 (disorder)	2202	9.7%
C-82423001	Chronic pain	752	3.3%
C-53741008	Coronary Heart Disease	1815	8.0%
C-44054006	Diabetes	2982	13.1%
C-55822004	Hyperlipidemia	4488	19.7%
C-302870006	Hypertriglyceridemia (disorder)	3151	13.8%
C-64859006	Osteoporosis (disorder)	1157	5.1%
C-15777000	Prediabetes	7272	31.9%
C-36971009	Sinusitis (disorder)	1220	5.3%

Table S6. Baseline characteristics of Synthea patients selected for the building lung cancer risk prediction ML model. The populate includes both patients with or without stroke. Code: standard codes prefixed with “C-”.

Comparison of ROC curves for lung cancer risk prediction XGBoost base models built from populations of 30K patients and 150K patients:

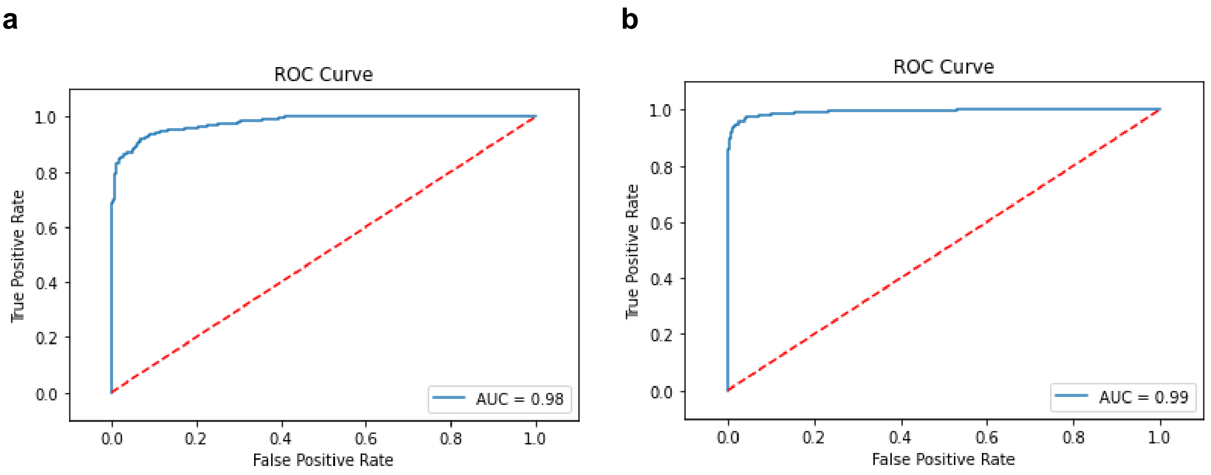

Fig S1. ROC curves for lung cancer risk prediction XGBoost base models. (a) Initial model built from pt30k dataset with 30K patients and 57 variables. **(b)** Updated model built from pt150k dataset with 150K patients and 137 variables.

Optimization of Lung Cancer XGBoost risk models

A 10-fold cross validation using the GridSearchCV() method found the following optimized parameters:

- scale_pos_weight: 3
- e_estimators: 200
- max_depth: 3
- eta: 0.1
- gamma: 0
- reg_lambda: 1.0

a

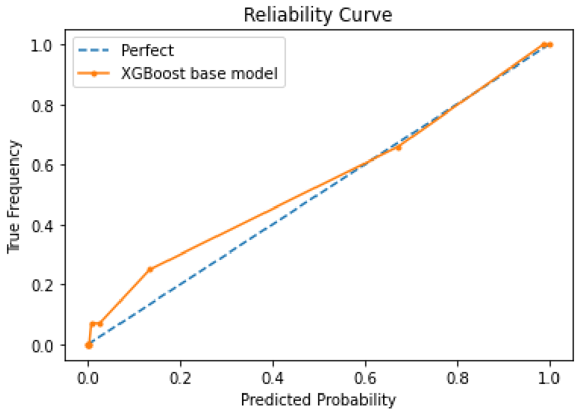

b

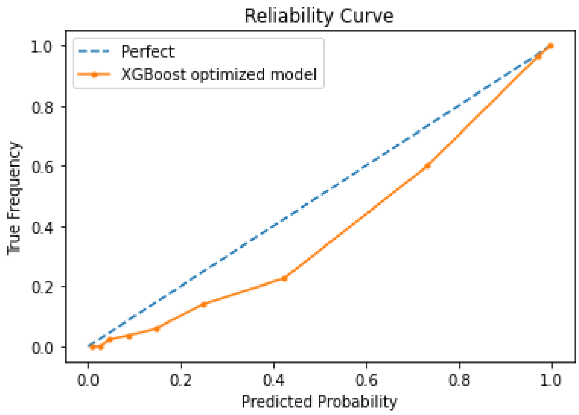

Fig S2. Reliability Curves of XGBoost models for 30K-patient dataset (pt30k). (a). Base model: recall = 0.849. **(b).** Optimized model: recall = 0.903.

Verification of the LHS process with another target disease stroke

Update Dataset	Total Patients	Target Patients	Target %	PDJ Data	BG Data	Selected Patients	Resample Target %
pt30k	30945	4125	13.3	183115	1693135	16055	25.7
pt60k	61900	8197	13.2	363246	3386667	32034	25.6
pt90k	92908	12251	13.2	542347	5077272	47908	25.6
pt120k	124170	16427	13.2	729091	6784032	64112	25.6
pt150k	155270	20494	13.2	910226	8490433	80266	25.5

Table S7. **Updated datasets for stroke risk models in the LHS.** Target patient percentage increases after resampling the imbalanced datasets. Target: stroke. BG: background. Selected patients: patients with or without stroke.

Code	Characteristics	Patient Count	Patient Percentage
	All	80266	100.0%
C-424144002	Age	80266	100.0%
	<50	21007	26.2%
	>=50	59259	73.8%
C-263495000	Gender	80266	100.0%
	Male	40616	50.6%
	Female	39650	49.4%
C-125680007	Marital status	80206	99.9%
	Married	64027	79.8%
	Single	16179	20.2%
C-103579009	Race	80266	100.0%
	White	65966	82.2%
	Black	6633	8.3%
	Asian	5492	6.8%
	Hawaiian	932	1.2%
	Native	360	0.4%
	Other	883	1.1%
C-186034007	Ethnicity	80266	100.0%
	Hispanic	8886	11.1%
	Nonhispanic	71380	88.9%
C-39156-5	Body Mass Index	70278	87.6%
	Abnormal	68585	85.4%
	Normal	1693	2.1%
C-72166-2	Tobacco smoking status NHIS	70278	87.6%
	Former	26870	33.5%
	Never	43408	54.1%
C-10509002	Acute bronchitis (disorder)	26342	32.8%
C-26929004	Alzheimer's disease (disorder)	1723	2.1%
C-271737000	Anemia (disorder)	25230	31.4%
C-49436004	Atrial Fibrillation	4394	5.5%
C-431855005	Chronic kidney disease stage 1 (disorder)	4141	5.2%
C-82423001	Chronic pain	2442	3.0%
C-53741008	Coronary Heart Disease	6494	8.1%
C-44054006	Diabetes	6972	8.7%
C-55822004	Hyperlipidemia	15207	18.9%
C-302870006	Hypertriglyceridemia (disorder)	7353	9.2%
C-64859006	Osteoporosis (disorder)	4823	6.0%
C-68496003	Polyp of colon	11383	14.2%
C-15777000	Prediabetes	24673	30.7%
C-36971009	Sinusitis (disorder)	3721	4.6%

Table S8. Baseline characteristics of Synthea patients selected for building stroke risk prediction ML model. This population includes both patients with or without stroke. Code: standard codes prefixed with "C-".

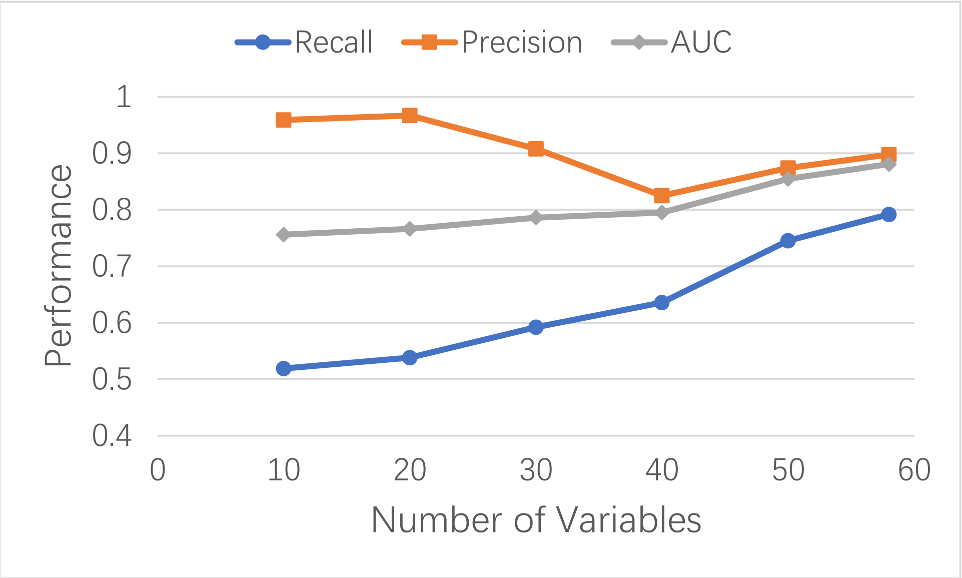

Fig S3. Trend of stroke risk prediction performance with increase in variable number. The initial XGBoost base models were built from the 30K-patient dataset.

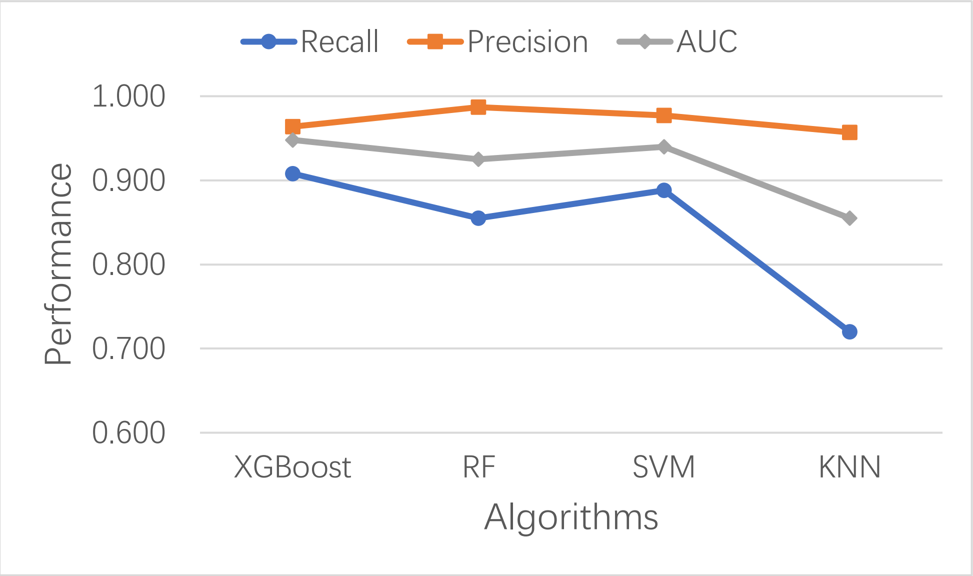

Fig S4. Comparison of the stroke risk prediction performance among XGBoost, RF, SVM and KNN base models. Models were based on a dataset of 150K patients with 124 variables.

a b

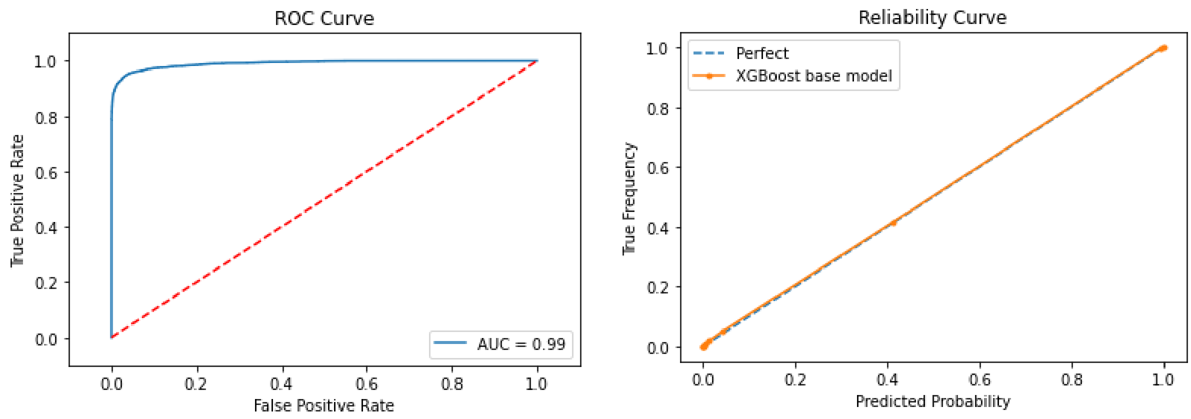

Fig S5. ROC and reliability curves of stroke risk prediction XGBoost base model built from the 150K-patient dataset (pt150k). (a) ROC curve. **(b)** Reliability curve, Brier score: 0.024.
